# Integration of Multi-Modal Biosensing Approaches for Depression: Current Status, Challenges, and Future Perspectives

**DOI:** 10.3390/s25154858

**Published:** 2025-08-07

**Authors:** Xuanzhu Zhao, Zhangrong Lou, Pir Tariq Shah, Chengjun Wu, Rong Liu, Wen Xie, Sheng Zhang

**Affiliations:** 1 Key Laboratory of Integrated Circuit and Biomedical Electronic System, Faculty of Medicine, Dalian University of Technology, Dalian 116024, China; xuanzhuzhao@163.com (X.Z.); louzr@dlut.edu.cn (Z.L.); pt.shah92@gmail.com (P.T.S.); xiewen001023@163.com (W.X.); 2 School of Health and Life Sciences, Qingdao Central Hospital, University of Health and Rehabilitation Sciences, Qingdao 266113, China; wcj5532@126.com; 3 Liaoning Key Lab of IC&BME System, School of Biomedical Engineering, Dalian University of Technology, No. 2 Linggong Street, Ganjingzi District, Dalian 116024, China; rliu@dlut.edu.cn; 4 State Key Laboratory of Fine Chemicals, Frontier Science Center for Smart Materials, School of Chemical Engineering, Dalian University of Technology, Dalian 116024, China

**Keywords:** biosensors, depression, multi-modal integration, temporal dynamics, wearable technologies

## Abstract

Depression represents one of the most prevalent mental health disorders globally, significantly impacting quality of life and posing substantial healthcare challenges. Traditional diagnostic methods rely on subjective assessments and clinical interviews, often leading to misdiagnosis, delayed treatment, and suboptimal outcomes. Recent advances in biosensing technologies offer promising avenues for objective depression assessment through detection of relevant biomarkers and physiological parameters. This review examines multi-modal biosensing approaches for depression by analyzing electrochemical biosensors for neurotransmitter monitoring alongside wearable sensors tracking autonomic, neural, and behavioral parameters. We explore sensor fusion methodologies, temporal dynamics analysis, and context-aware frameworks that enhance monitoring accuracy through complementary data streams. The review discusses clinical validation across diagnostic, screening, and treatment applications, identifying performance metrics, implementation challenges, and ethical considerations. We outline technical barriers, user acceptance factors, and data privacy concerns while presenting a development roadmap for personalized, continuous monitoring solutions. This integrative approach holds significant potential to revolutionize depression care by enabling earlier detection, precise diagnosis, tailored treatment, and sensitive monitoring guided by objective biosignatures. Successful implementation requires interdisciplinary collaboration among engineers, clinicians, data scientists, and end-users to balance technical sophistication with practical usability across diverse healthcare contexts.

## 1. Introduction

Depression represents one of the most prevalent and debilitating mental health disorders worldwide, affecting more than 280 million people across all age groups and socioeconomic backgrounds as reported by the World Health Organization, with approximately 3.8% of the global population experiencing depression [1,2]. Despite its prevalence and impact, depression remains challenging to diagnose and treat effectively. Current clinical practices rely predominantly on subjective assessments and episodic evaluations that fail to capture the dynamic, multifaceted nature of this disorder [3,4].

The complex etiology and manifestation of depression involve interactions across multiple biological systems, including neurotransmitter imbalances [5], hypothalamic–pituitary–adrenal axis dysregulation [6], autonomic nervous system dysfunction [7], altered sleep architecture [8,9], disrupted circadian rhythms [10,11], and neuroinflammatory processes [12]. This multisystem involvement produces heterogeneous symptom profiles that evolve dynamically, with significant variability in presentation, progression, and treatment response [13]. Such complexity necessitates comprehensive monitoring approaches capable of capturing diverse biological signals and behavioral patterns across different contexts and timeframes.

Current diagnostic and monitoring approaches face fundamental limitations, including dependence on subjective assessments, episodic evaluation constraints, contextual information gaps, and biological marker limitations [4]. Recent advances in biosensing technologies, however, offer unprecedented opportunities for objective, quantitative assessment of depression-relevant biomarkers and physiological parameters [14]. These technologies, from electrochemical detection of neurotransmitters to wearable monitoring of physiological and behavioral patterns, provide promising avenues for enhancing depression diagnosis and treatment optimization [15,16].

**Figure 1:**
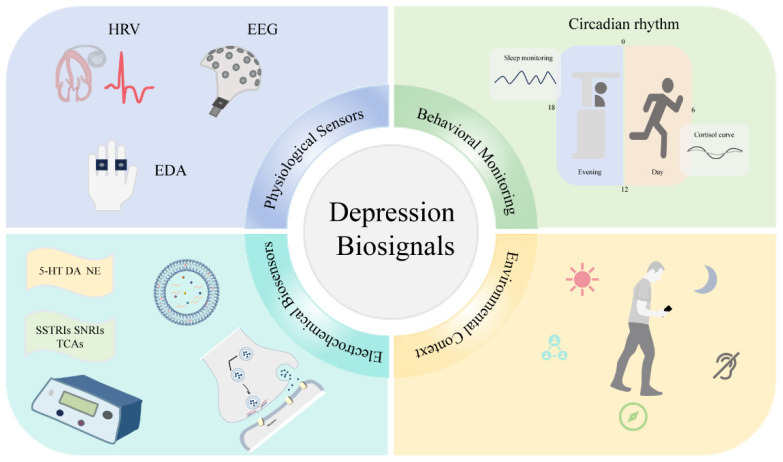
Conceptual framework of multi-modal biosensing for depression.

The concept of precision psychiatry has gained significant momentum, with technological advances providing essential implementation tools. Multi-modal biosensing approaches represent key enabling technologies for this paradigm by providing objective, personalized measurements that can guide individualized care decisions [17,18]. By capturing diverse biological and behavioral signals across contexts and timeframes, these approaches may eventually support more precise depression subtyping, treatment selection, and outcome monitoring than conventional methods alone [19]. This review examines the current landscape of multi-modal biosensing for depression, focusing on technological capabilities, integration strategies, and clinical applications while identifying key challenges and future directions in this rapidly evolving field, as illustrated in Figure 1.

## 2. Current State of Biosensor Technologies for Depression

**Figure 2:**
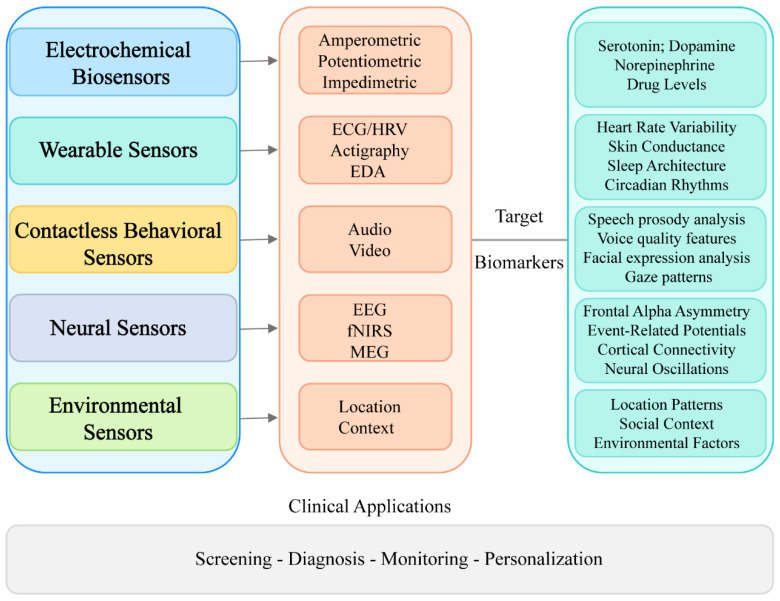
Classification of biosensors for depression monitoring.

Depression monitoring biosensors comprise a range of technological approaches that target specific biological and behavioral parameters associated with depressive states. Figure 2 illustrates a hierarchical classification of these technologies, demonstrating their progression from fundamental sensing modalities to clinical applications. The framework categorizes biosensors into five principal groups, e.g., electrochemical, wearable, neural, behavioral, and environmental, each featuring distinctive subcategories and corresponding biomarkers. This taxonomic structure provides critical context for assessing the comparative strengths, limitations, and complementary features of various sensing methodologies in depression evaluation.

### 2.1. Electrochemical Biosensors

Electrochemical biosensors represent one of the most extensively developed platforms for depression biomarker detection, particularly for neurotransmitters and related molecules [20,21]. These devices convert biochemical interactions into measurable electrical signals via techniques such as amperometry, voltammetry, impedimetry, and potentiometry [22]. The integration of nanomaterials with electrochemical biosensors has significantly enhanced their capabilities for neurological disorder monitoring, providing critical tools for precise neurotransmitter detection [23] with potential applications in both clinical diagnosis and treatment management.

Studies have demonstrated the clinical utility of these aforementioned sensors. Researchers developed carbon nanotube-implanted polymer micropillar electrodes achieving dopamine detection limits of 0.435 nM via cyclic voltammetry, with differential pulse voltammetry yielding quantification limits of 2.34 nM and sensitivity of 0.453 μA/μM [24]. Machine learning has enhanced serotonin detection, as demonstrated in using DNA-wrapped single-walled carbon nanotube sensors with near-infrared fluorescence spectroscopy to identify optical response sequences providing superior enzyme-specific serotonin responses compared to conventional probes [25]. These advances align with clinical needs identified in [26], which highlight bioreceptors, including nucleic acids, proteins, and cellular elements, as essential components for medical electrochemical biosensing. This clinical relevance is further demonstrated by [27], which advocates for expanded implementation of therapeutic drug monitoring for antidepressants in primary care settings to optimize treatment efficacy and safety, noting that such monitoring remains significantly underutilized despite its proven benefits.

Significant progress has been made in developing electrochemical biosensors for key depression-related neurotransmitters, including serotonin, dopamine, and norepinephrine [21]. Carbon-based nanomaterials, particularly graphene derivatives and carbon nanotubes, have markedly improved serotonin and dopamine detection through enhanced electron transfer kinetics, greater surface area, and three-dimensional conducting networks that amplify signals while facilitating biomolecule immobilization [28]. Additionally, the incorporation of advanced materials such as gold nanoparticles and metal-organic frameworks has further enhanced sensor performance, enabling detection at clinically relevant concentrations [29].

Beyond neurotransmitters, electrochemical biosensors have been developed for monitoring antidepressant drug levels, including selective serotonin reuptake inhibitors (SSRIs), serotonin-norepinephrine reuptake inhibitors (SNRIs), and tricyclic antidepressants [21]. These electrochemical biosensors offer significant advantages for therapeutic drug monitoring through their ability to detect trace amounts of antidepressants in various biological samples with high specificity and sensitivity, potentially enabling personalized medication dosing strategies and improved treatment outcomes [30]. Surface-modified electrodes using specialized nanomaterials have dramatically improved detection capabilities, overcoming challenges such as electrode fouling and interference from other biomolecules in complex biological matrices [31].

These antidepressant monitoring systems address fundamental clinical needs by supporting personalized medication management. The electrochemical sensors provide rapid, accurate measurements that may facilitate treatment optimization, particularly for patients experiencing adverse reactions or inadequate therapeutic responses. This approach holds promise for point-of-care testing that could transform depression management through more precise therapeutic drug monitoring.

### 2.2. Wearable Physiological Sensors

Wearable devices capable of continuous monitoring of physiological parameters have emerged as valuable tools for depression assessment [32]. These technologies enable non-invasive, longitudinal monitoring in natural environments, potentially capturing temporal patterns and contextual factors relevant to depression [33]. Recent advances in sensor miniaturization, data processing algorithms, and wireless connectivity have significantly enhanced the clinical utility of these devices [34].

Electroencephalography (EEG)-based wearables, which measure brain electrical activity, have demonstrated considerable promise for depression detection, with advanced machine learning algorithms yielding classification accuracies of 90% in discriminating between depressed and non-depressed individuals [35]. While traditional clinical EEG systems require conductive gels and complex preparation procedures, newer consumer-grade devices with fewer electrodes and dry electrode technology offer greater accessibility and comfort for extended wear [36]. Several commercial EEG wearables have emerged with varying capabilities for mental health monitoring. Consumer-grade devices such as the Muse S headband (4 electrodes) have demonstrated competitive performance compared to traditional 32-channel EEG setups for emotion detection, particularly in the gamma band for valence detection, while the Emotiv EPOC Flex has been validated for EEG and ERP research applications [37,38,39]. High-density dry electrode EEG systems have demonstrated feasibility for real-time neuroimaging and cognitive monitoring, with performance comparable to traditional wet electrode systems for measuring EEG spectra and ERP components [40,41]. Consumer-grade EEG devices, including the NeuroSky MindWave Mobile 2 (single dry electrode, 512 Hz sampling rate), have been extensively validated for research applications [42]. While these devices are marketed for wellness and research purposes, no consumer-grade EEG device currently holds FDA approval specifically for depression diagnosis. In Europe, consumer EEG devices including the Emotiv systems have obtained CE marking, indicating compliance with safety standards for their intended use [39]. Although these consumer devices typically show reduced accuracy compared to medical-grade systems, recent innovations have significantly narrowed this performance gap, particularly through transformer-based frameworks that leverage both temporal and spatial characteristics of EEG data linked to depression [43] and through the integration of multiple sensor types for improved diagnostic capability [44].

Heart rate variability (HRV) and electrocardiogram (ECG) sensors provide critical insights into autonomic nervous system function, which is frequently dysregulated in depression, as evidenced by the reduced HRV commonly observed in affected individuals [45]. By measuring the fluctuation in time between heartbeats, HRV reflects the balance between sympathetic and parasympathetic nervous system activity, with multiple studies identifying specific patterns associated with depression, particularly decreased parasympathetic activity measured through high-frequency HRV components [46]. Research demonstrates that reduced overall HRV correlates with depression symptom severity, suggesting its potential utility as a biomarker for affective disorders [47]. Recent technological advances have significantly improved accessibility to HRV monitoring through wearable devices that enable long-term tracking in non-clinical environments, providing more comprehensive data than traditional short-term clinical recordings [48]. Furthermore, deep learning algorithms applied to HRV data collected from wrist-worn devices have shown promising results in predicting various mental health outcomes, including depression, stress, and anxiety levels, thus enhancing the clinical applicability of these technologies [49].

Actigraphy and movement sensors provide objective measures of psychomotor changes and activity patterns that strongly correlate with depression severity [50], detecting subtle alterations in movement, sleep quality, and circadian rhythms that subjective reporting often fails to capture and that reflect core depression features such as psychomotor retardation and reduced daily activity [51]. Research has consistently demonstrated that individuals with depression exhibit distinctive activity patterns compared to healthy controls, with significant differences in daily activity metrics and sleep-related measurements that often improve following successful treatment [52]. The diagnostic potential of these technologies has been substantially enhanced by recent machine learning approaches, with one study achieving remarkable 98% accuracy and F1-scores by combining actigraphy data with demographic and clinical information through Random Forest, AdaBoost, and Artificial Neural Network algorithms [53,54], while a comprehensive systematic review and meta-analysis reported pooled mean accuracies of 70–89% for depression detection using wearable devices, underscoring the significant clinical utility of actigraphy in both research and clinical settings [15].

Systematic reviews of wearable artificial intelligence for depression detection have demonstrated impressive clinical potential, with pooled mean values for accuracy, sensitivity, and specificity of 0.89, 0.87, and 0.93, respectively [15], while the most advanced multi-modal systems combining ECG, EDA, and respiration measurements have achieved remarkably high accuracies exceeding 99% in controlled laboratory settings, as reported in a study using the WESAD dataset [55]. These wearable technologies offer unique advantages by capturing physiological signals that conventional monitoring methods cannot detect, including critical sleep-related information and real-time measurements of heart rate, skin temperature, and energy expenditure, all parameters strongly associated with depressive states [56,57]. Unlike smartphone-based monitoring, wearable devices provide continuous physiological data collection, enabling comprehensive assessment of subtle physiological changes indicative of depression onset or progression [58]. Table 1 compares the different biosensor technologies discussed above, including their target biomarkers, strengths, and limitations. The integration of these technologies into clinical practice promises to transform depression management through earlier detection, more objective assessment, and personalized treatment approaches, though further validation studies in diverse populations and real-world environments remain essential to establish their reliability and maximize their clinical utility across different patient groups and healthcare settings.

### 2.3. Contactless Behavioral Sensors

Contactless sensing through audio and video analysis has emerged as a powerful approach for depression assessment, offering non-invasive, naturalistic monitoring without physical devices or active user engagement. These technologies harness advances in signal processing, computer vision, and machine learning to extract behavioral and acoustic markers of depression from speech and visual data. By complementing traditional biosensors while reducing user burden, contactless methods enable scalable deployment across diverse clinical and real-world settings.

Speech and voice characteristics serve as valuable depression biomarkers, reflecting psychomotor, cognitive, and affective disruptions through multiple acoustic dimensions [63]. Prosodic analysis reveals reduced pitch variability, slower speaking rates, prolonged pauses, and diminished vocal intensity in depressed speech [64], while voice quality measures capture increased roughness, breathiness, and strain through parameters such as jitter, shimmer, and harmonics-to-noise ratio [65]. Advanced signal processing extracts depression-relevant features including fundamental frequency contours, formant frequencies, mel-frequency cepstral coefficients, and spectral energy distributions [66], alongside temporal dynamics such as altered speech rhythm, voice onset times, and conversational turn-taking patterns [67]. Beyond diagnostic applications, longitudinal speech analysis shows promise for monitoring treatment response and predicting relapse risk, offering objective measures that complement traditional clinical assessments.

Computer vision-based behavioral analysis captures depression-related changes across multiple non-verbal channels, with deep learning advances dramatically improving feature extraction accuracy [68]. Facial expression analysis reveals reduced emotional expressivity, particularly for positive affect, with Action Unit detection identifying specific patterns such as decreased AU12 (lip corner puller) and increased AU15 (lip corner depressor) activity [69]. Dynamic facial analysis captures temporal features including expression timing, intensity variations, and symmetry measures, while 3D modeling and micro-expression detection enhance sensitivity to subtle affective changes [70]. Gaze behavior analysis demonstrates altered visual scanning patterns characteristic of depression, including reduced exploratory movements, prolonged fixations on negative stimuli, and diminished eye contact, now measurable through video-based estimation without specialized hardware [71,72]. Additionally, computer vision algorithms quantify psychomotor manifestations through head pose dynamics, movement velocity profiles, and whole-body pose estimation, revealing reduced gesture amplitude, decreased movement variability, and altered social proxemics that correlate with clinical ratings of psychomotor retardation [73,74]. These integrated visual behavioral markers show particular promise for assessing depression severity and monitoring treatment response in naturalistic settings.

### 2.4. Neural Sensors

Neural sensing technologies directly measure depression-related brain activity, revealing neurophysiological mechanisms that complement peripheral biosensing. Electroencephalography (EEG), magnetoencephalography (MEG), and functional near-infrared spectroscopy (fNIRS) show particular promise for depression assessment given their excellent temporal resolution and non-invasiveness. EEG and fNIRS offer additional advantages of portability and low cost. These technologies enable the transition from laboratory neuroimaging to real-world monitoring, facilitating longitudinal assessment of neural biomarkers for predicting treatment response and tracking symptom progression.

Neurophysiological assessment of depression leverages complementary imaging modalities that capture distinct aspects of neural dysfunction. EEG reveals established biomarkers including frontal alpha asymmetry, reflecting reduced approach motivation, and altered power spectral density characterized by increased theta/beta activity with decreased frontal alpha, patterns that correlate with symptom severity and normalize following successful treatment [75]. Advanced signal processing and machine learning achieve >90% classification accuracy [76], while functional connectivity analyses demonstrate network-level disruptions including reduced interhemispheric coherence and impaired information integration within default mode and frontoparietal networks [77]. MEG provides superior spatiotemporal resolution for detecting disrupted oscillatory activity, particularly gamma-band alterations (30–80 Hz) that correlate with cognitive symptoms and treatment response, with source localization revealing prefrontal and anterior cingulate hypoactivity during emotional processing. Pharmaco-magnetoencephalography uniquely tracks rapid neural changes following ketamine administration, revealing gamma-band alterations that precede clinical improvement [78]. fNIRS complements these approaches by measuring cortical hemodynamic responses, consistently demonstrating prefrontal hypoactivation during cognitive and emotional tasks, with slower response functions and reduced interhemispheric connectivity in depressed individuals [79,80]. While MEG remains limited to specialized settings due to cost and environmental constraints, recent advances in portable EEG systems with dry electrodes and optically pumped magnetometers, combined with multi-modal integration approaches, promise enhanced clinical translation of these neurophysiological biomarkers for depression assessment and treatment monitoring.

### 2.5. Environmental Sensors

Environmental factors significantly influence depression onset, maintenance, and recovery, with evidence linking ambient conditions, circadian disruption, and spatial behaviors to mood regulation. Continuous monitoring through environmental sensors, capturing light exposure, temperature variations, and location patterns, provides objective measures of how individuals interact with their surroundings, complementing traditional biological and behavioral assessments. These technologies reveal previously inaccessible patterns of person–environment interaction, demonstrating how environmental factors moderate both depression symptoms and treatment response, thereby advancing understanding of contextual influences on mood disorders.

Environmental and behavioral monitoring technologies provide multi-modal insights into depression pathophysiology through continuous assessment of light exposure, ambient conditions, and mobility patterns. Wearable sensors reveal that depressed individuals experience reduced daytime bright light exposure, particularly deficient morning blue light (480 nm) that influences circadian regulation via melanopsin pathways, alongside increased evening exposure, patterns that correlate with circadian phase delays, rhythm amplitude reduction, and symptom severity [81,82]. Environmental monitoring extends beyond light to demonstrate blunted circadian temperature rhythms, impaired thermoregulation, and heightened sensitivity to indoor air quality parameters including CO_2_ and volatile organic compounds, with these factors interacting to influence mood through effects on sleep, activity, and social behavior [83,84]. GPS and positioning technologies complement physiological monitoring by revealing behavioral manifestations of depression including reduced location variance, prolonged home stays, irregular routines, and social isolation reflected in public space avoidance, mobility metrics that correlate with clinical ratings and treatment response [85]. Machine learning models integrating these multi-modal features achieve 80–85% accuracy in detecting depressive episodes, with performance enhanced by incorporating geospatial context such as green space access and neighborhood characteristics [86,87]. Privacy-preserving edge computing enables real-time pattern detection while longitudinal analysis reveals that environmental and behavioral changes often precede mood shifts by days, suggesting opportunities for early intervention through both environmental optimization and targeted therapeutic strategies that address the complex interplay between circadian disruption, environmental sensitivity, and behavioral withdrawal in depression.

## 3. Multi-Modal Integration Approaches

### 3.1. Sensor Fusion Strategies

**Figure 3:**
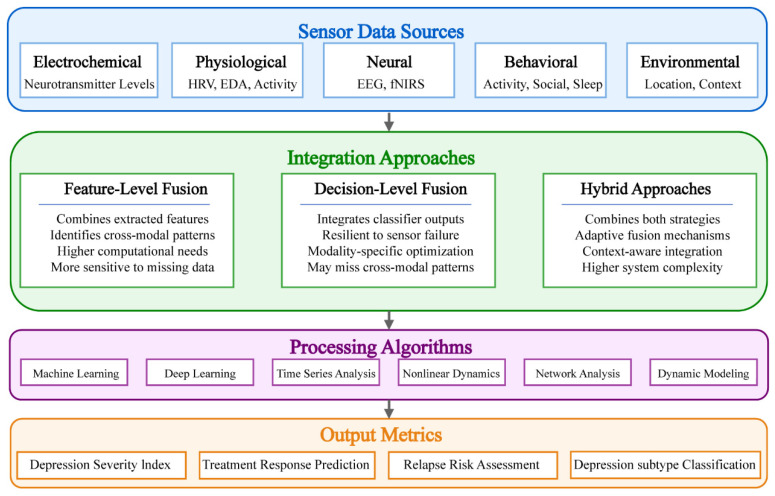
Sensor fusion strategies for depression monitoring.

Depression’s complex, multifaceted nature necessitates comprehensive monitoring approaches that capture diverse biological systems and phenomenological manifestation. No single biomarker adequately represents this complexity, underscoring the need for integrated, multi-modal strategy [88]. As illustrated in Figure 3, multi-modal sensor fusion has emerged as a powerful framework for integrating depression-relevant biosignals, accommodating heterogeneous data types, sampling frequencies, and reliability characteristics [89]. Feature-level fusion combines extracted features before classification, enabling identification of cross-modal patterns [90], while decision-level fusion integrates independent modality-specific predictions, providing resilience against sensor failure [91]. Hybrid approaches combining both strategies have demonstrated promising experimental results [92,93]. Recent advances in dynamic fusion techniques accommodate fluctuating data availability and quality, representing a frontier for ecological depression monitoring [94]. These technological developments significantly enhance depression detection accuracy and treatment monitoring capabilities in a real-world environment.

### 3.2. Temporal Dynamics and Rhythm Analysis

Depression represents a dynamic condition with fluctuations across multiple timescales, from moment-to-moment mood variations to episodic recurrences spanning months or years [95]. Multi-modal biosensing provides unprecedented opportunities to characterize these temporal dysregulations and their relationship to depression symptomatology [17].

Depression’s temporal dynamics manifest across biological and behavioral rhythms, with diurnal pattern analysis revealing characteristic signatures through 24 h neurochemical, physiological, and behavioral patterns. These analyses identify distinct “chronotypes”—individual differences in circadian rhythm preferences (e.g., morning versus evening types)—with varying treatment responses, as depression is often associated with disrupted circadian clock-controlled responses like sleep and cortisol secretion [96,97]. Concordance between cortisol rhythms and behavioral patterns predicts outcomes more accurately than single-domain measures, with evening-type individuals showing significantly greater vulnerability to depression compared to morning-types [98]. Studies have found sex differences in these chronotype–depression associations, with women with late chronotype showing a higher risk of depression after adjusting for covariates [99]. Treatment-resistant depression exhibits weakened coupling between ultradian cortisol pulses and activity patterns, as the temporal dynamics of cortisol may be altered in depression across different timescales [100].

Nonlinear dynamics analysis of integrated multi-modal data reveals reduced complexity characteristic of depression, with investigations showing that nonlinear indices better capture relationships between heart-rate dynamics, cognition, and mood than traditional time and frequency measures [101]. Multiscale entropy analysis, a method that quantifies signal complexity across multiple timescales to characterize system dynamics, of cardiovascular, activity, and sleep data demonstrates diminished complexity across timescales, particularly in physiological–behavioral coupling domains, a finding consistent with the theory of complexity-loss with disease [102]. Sleep EEG analysis using nonlinear methods has shown that complexity decreases from wakefulness to deeper sleep stages, with depression associated with disruptions in these normal entropy patterns [103]. The application of multiscale entropy to cardiac rhythm data identifies autonomic dysfunction in depression, with nonlinear analysis demonstrating the loss of normal circadian change in patients [104,105].

Network analysis of biosignals unveils functional connectivity abnormalities in major depressive disorder, reflecting underlying neural dysregulation [106]. Neurophysiological depression subtypes, characterized by distinct limbic–frontostriatal connectivity patterns, facilitate precise diagnostic classification [107]. Critical differentiation between depression variants emerges from network transitional dynamics, particularly neural reconfiguration adaptability to environmental challenges [108]. This multi-modal, multiscale temporal analysis approach provides a compelling framework for depression characterization, subtyping, and treatment optimization.

### 3.3. Contextual Awareness and Environmental Integration

Environmental and situational contexts modulate depression-relevant biosignals, requiring context-aware analytical approaches for meaningful interpretation [109]. Ecological momentary assessment combined with passive biosensing enhances depression detection by accounting for context-dependent variability in physiological and behavioral parameters [110]. Environmental context modeling links geographic data with multi-modal sensing to show how urban density, green space, and ambient noise influence relationships between mobility patterns and depression severity [111,112]. Access to high-quality natural environments correlates with reduced depression risk through community belonging and improved stress recovery mechanisms [113]. Socioeconomic disparities in urban green space distribution may exacerbate mental health inequalities across populations [114].

Social contexts play a crucial role in interpreting manifestations of depression, as integrated sensor technologies have revealed distinctive behavioral patterns in individuals with depression during social interactions that often remain undetectable in non-social environments [115,116]. Social proximity features, metrics derived from smartphone sensors including Bluetooth, GPS, and communication logs, when analyzed alongside environmental context, provide informative biomarkers of depressive symptomatology, with increased mobility correlating with symptom improvement [117,118]. Furthermore, analysis of ambient audio recordings from naturalistic environments yields valuable sociability metrics that effectively characterize depressive states [119]. Research shows robust associations between depression severity and quantifiable vocal social behaviors, including conversation frequency, duration, and characteristics of the acoustic environment [116,120].

Adaptive context-sensitive algorithms improve ecological validity by adjusting physiological parameter interpretations based on context (activity, posture, social setting), enabling more precise depression monitoring [121,122]. Machine learning applied to multi-modal sensor data (audio, proximity, physiological) enhances screening, complements clinical assessments, and tracks symptoms between visits [115,123]. Combining wearable, environmental, and smartphone passive sensing captures comprehensive physiological and behavioral patterns across varied contexts [57,124].

### 3.4. Case Studies of Multi-Modal Integration

Multi-modal biosensing has emerged as a powerful approach for enhancing accuracy, reliability, and clinical utility of mental health monitoring systems, with evidence from major research initiatives demonstrating this potential across diverse clinical contexts [44,125]. The FAITH project utilizes wearable sensors, smartphone usage patterns, and structured psychological assessments within a federated learning architecture, a privacy-preserving machine learning approach that trains algorithms across decentralized data without sharing raw patient information, to monitor depressive symptoms in cancer survivors while preserving patient privacy [126]. Extending this approach to multiple neuropsychiatric conditions, the RADAR-CNS initiative has achieved significantly improved detection of depressive episodes compared to unimodal approaches, maintaining high data completion rates throughout longitudinal studies [127]. Complementary physiological signal integration, particularly electroencephalography (EEG) with heart rate variability (HRV), has advanced through sophisticated analytical methods such as hierarchical feature fusion and multi-kernel learning, yielding substantial accuracy improvements by capturing complex neural–cardiac interactions [128]. Beyond traditional physiological measurements, digital phenotyping has expanded multi-modal applications, exemplified by smartphone-based studies combining sensing, sleep metrics, and cognitive assessments to achieve high classification accuracy for major depressive disorder [129], while ecological momentary assessment approaches integrating physical activity data with psychological measurements demonstrate superior assessment capabilities [130]. These findings underscore how strategic integration of complementary data streams enhances mental health assessment precision while maintaining implementation feasibility, though challenges in data synchronization and cross-platform standardization necessitate continued investigation.

### 3.5. Performance Metrics Summary

To facilitate direct comparison of the various biosensing approaches discussed in this review, Table 2 presents a comprehensive summary of key performance metrics, including accuracy, sensitivity, specificity, and device characteristics. This synthesis enables readers to quickly assess the relative strengths and trade-offs of different monitoring modalities for depression assessment.

## 4. Clinical Applications and Validation

**Figure 4:**
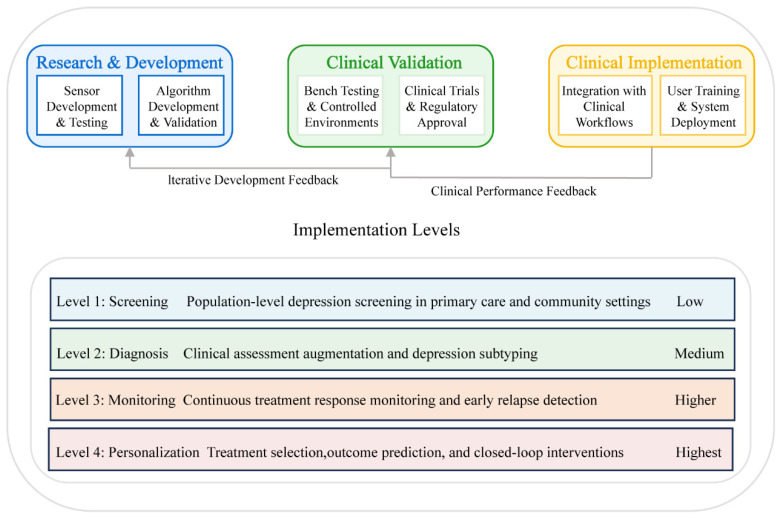
Clinical implementation and validation pathway.

Implementing biosensing technologies for depression in clinical settings necessitates a structured validation and integration pathway to ensure both technological efficacy and practical utility. Figure 4 illustrates a four-tiered implementation framework of increasing complexity, ranging from population-level screening to personalized intervention approaches. These tiers represent progressive levels of technological sophistication and clinical integration, establishing a continuum from basic assessment to precision treatment. This stratified approach enables healthcare environments to incorporate biosensing technologies at levels appropriate to their available resources, clinical expertise, and specific patient populations.

### 4.1. Diagnostic and Screening Applications

Multi-modal biosensing has emerged as a promising approach as an adjunct to traditional clinical assessment for depression screening and diagnosis [68,131]. Integrated systems combining smartphone-derived behavioral biomarkers with physiological parameters from wearable devices have demonstrated robust diagnostic performance metrics, including accuracy up to 89%, sensitivity of 87%, and specificity of 93% when evaluated under rigorous experimental conditions [15]. Initial validation studies suggest potential for improving mental healthcare access in underserved areas; however, addressing reliability, user acceptance, privacy, and cost barriers remains essential for widespread clinical adoption [132,133].

Recent studies have investigated applications for differential diagnosis in which integrated behavioral, autonomic, and neuroimaging features differentiate bipolar from unipolar depression with 73–80% accuracy, although comprehensive clinical validation remains limited [134,135]. In terms of severity assessment, these multi-modal systems demonstrate moderate correlations (r = 0.5–0.7) with established clinician-rated scales and exhibit promising potential for longitudinal symptom monitoring, potentially offering significant advantages over conventional assessment methods [136].

Despite promising findings across the depression care continuum, substantial implementation barriers continue to exist regarding data integration, incorporation into clinical workflows, and validation across diverse populations, highlighting the necessity for larger and more heterogeneous cohort studies before widespread clinical implementation. Ongoing interdisciplinary development of these technologies may significantly enhance depression management through objective, longitudinal assessments that complement clinical expertise, while important ethical considerations concerning data privacy, algorithmic bias, and appropriate technology utilization remain critical as these approaches evolve toward clinical maturity.

### 4.2. Treatment Monitoring and Outcome Prediction

Multi-modal biosensing presents significant potential for continuous, objective monitoring of treatment response in depression, addressing inherent limitations of conventional episodic assessments [137]. Integrated analyses of autonomic nervous system functioning, physical activity patterns, sleep architecture, and social interaction metrics during the initial phase of antidepressant therapy demonstrate promising predictive capability for treatment outcomes, with reported accuracy rates ranging from 65% to 80%, surpassing predictions derived solely from clinical features [138,139]. Furthermore, continuous monitoring systems show promise for detecting depression relapse, as biosignature changes may precede clinical symptoms by days to weeks, enabling preemptive interventions [138,140].

Beyond efficacy assessment, multi-modal biosensing approaches may facilitate personalized treatment selection through identified correlations between specific biosignature patterns and differential responses to various antidepressant modalities [141,142]. Early changes in physiological and behavioral parameters might distinguish outcomes between cognitive-behavioral therapy and pharmacological interventions, although this research direction remains in its exploratory stages [143]. Between scheduled clinical evaluations, longitudinal monitoring utilizing passive smartphone sensing technologies supplemented with periodic structured assessments may enhance depression management by tracking symptom fluctuations with potentially greater sensitivity than conventional monthly clinical evaluations [118].

Collectively, these technological advances suggest that multi-modal biosensing could fundamentally transform depression treatment paradigms, shifting from episodic clinical evaluations toward dynamic, personalized care pathways guided by objective biological and behavioral monitoring [17,144]. However, substantial validation across diverse clinical populations and treatment settings remains essential before widespread implementation can be recommended.

## 5. Technical and Implementation Challenges

**Figure 5:**
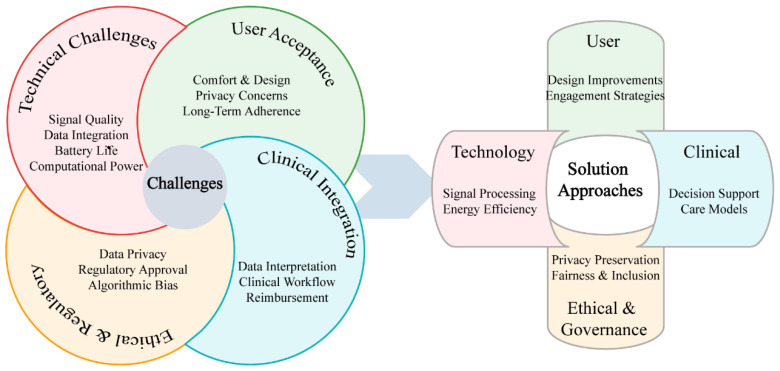
Technical and implementation challenges in multi-modal biosensing for depression.

Despite promising advances in multi-modal biosensing for depression, significant challenges must be addressed before widespread clinical implementation becomes feasible [145]. These challenges span technical limitations, practical implementation barriers, and ethical considerations that collectively influence the translation pathway from laboratory validation to real-world clinical utility, as illustrated in Figure 5.

### 5.1. Technical Challenges

Signal quality and reliability in naturalistic environments represent critical barriers to clinical adoption of biosensing technologies for depression monitoring. Despite promising laboratory performance, real-world conditions compromise measurement integrity through motion artifacts, electrode displacement, sweat-induced impedance changes, and electromagnetic interference. Wearable EEG systems suffer from muscle artifacts, electrode displacement, and electrical interference, with validation studies revealing error rates that substantially increase during movement compared to stationary conditions [146]. Similarly, wrist-worn photoplethysmography accuracy diminishes significantly during exercise compared to electrocardiogram standards, potentially undermining assessments of autonomic function critical for depression biomarkers [147,148]. Electrochemical biosensors for neurotransmitter and drug monitoring show significant sensitivity reductions in field studies due to temperature fluctuations, pH variations, and interfering substances in physiological fluids [16]. Although mitigation strategies such as advanced signal processing, sensor fusion, machine learning, and self-calibrating designs show promise, they introduce computational complexity that constrains real-time implementation in resource-limited wearable platforms. Persistent challenges in power consumption, processing latency, and algorithm robustness across diverse conditions continue to impede clinical deployment.

Sensor integration for depression monitoring faces multifaceted challenges stemming from the heterogeneous nature of biosignals. Temporal alignment difficulties arise when reconciling disparate timescales, from millisecond-level EEG fluctuations to multi-day sleep cycles, particularly when establishing causal relationships between rapid physiological changes and gradual behavioral indicators [149,150]. Data heterogeneity compounds these challenges as modalities generate fundamentally different data types (continuous waveforms, discrete events, binary states, and concentration values), necessitating sophisticated transformation approaches [151,152]. The integration of heterogeneous data streams from wearable technologies presents critical challenges requiring methodological approaches that address both technical aspects of sensor data fusion and ethical considerations of terminology and transparency in digital health monitoring systems [153,154]. While current mitigation strategies include time-windowing techniques, feature-level fusion, ontology-based modeling, and open-source middleware platforms, the development of unified standards remains constrained by commercial interests, insufficient regulatory frameworks, and technical complexity, with emerging standardization initiatives still limited in scope and adoption.

Battery life and computational power limitations significantly constrain continuous monitoring capabilities in wearable biosensors for depression. Physiological monitoring demands substantial power for signal acquisition, processing, and transmission, limiting continuous operation duration and thereby restricting the longitudinal data collection necessary for identifying relevant patterns [155]. Advanced analytics demand computational resources exceeding on-device capabilities [156], creating a challenging trade-off: cloud-based processing introduces connectivity dependencies, latency, and privacy concerns [157], while edge computing implementations face severe processing and memory constraints [158]. Storage requirements further complicate implementation, with comprehensive EEG datasets requiring capacity for tens of thousands of trials and dozens of recording hours [159], while processing such extensive neurophysiological data demands robust quality metrics and standardized assessment methods [160]. Current strategies to address these limitations include: (1) adaptive sampling, which optimizes acquisition parameters based on detected activity states [161]; (2) hierarchical processing architectures that conduct lightweight analysis on-device while selectively offloading complex computations; (3) compressive sensing algorithms that leverage signal sparsity to reduce data acquisition requirements [162]; and (4) energy harvesting from environmental sources such as motion, thermal gradients, or solar radiation [163]. Despite these innovations, harvesting technologies currently yield only modest power contributions, insufficient to sustain full operational requirements. While these approaches show promise in controlled settings, significant improvements in energy density, computational efficiency, and algorithm optimization remain necessary for enabling truly long-term continuous monitoring with sophisticated analytical capabilities.

### 5.2. Practical Implementation Barriers

User acceptance and adherence remain critical challenges for wearable biosensor implementation in depression monitoring, as even technically sophisticated systems provide limited value without consistent utilization [164]. Contemporary physiological monitoring technologies frequently utilize form factors that significantly impede routine activities. Electroencephalography (EEG) monitoring necessitates conspicuous head-worn devices that induce discomfort during extended use periods [165], whereas medical-grade electrocardiography (ECG) monitoring systems typically require chest-worn apparatus that restricts movement and constrains clothing options [166]. Many systems require technical expertise for proper setup and calibration that exceeds typical user capabilities, particularly problematic for older adults who experience higher rates of depression, with research demonstrating strong correlations between technical complexity and abandonment rates Older Adults Perceptions of Technology [167]. The “novelty effect” presents additional challenges, as wearable devices often exhibit high initial adoption rates followed by substantial attrition [168], particularly problematic for depression monitoring requiring consistent long-term data collection. Promising approaches addressing these challenges include user-centered design methodologies prioritizing comfort and discreet form factors; gamification strategies leveraging behavioral economics principles to enhance sustained engagement [169]; personalized feedback mechanisms providing actionable insights rather than raw data; and multi-form-factor ecosystems offering context-appropriate monitoring options [170].

Clinical workflow integration poses a significant barrier as healthcare systems lack protocols for incorporating continuous biosignal data into clinical practice. Multi-modal biosensing generates substantial volumes of data, potentially gigabytes weekly from a single patient, exceeding the processing capacity of time-constrained clinical encounters [44]. Most mental health providers, particularly psychiatrists and primary care physicians who treat the majority of depression cases, lack sufficient training to interpret complex biosignal patterns beyond basic activity and sleep measures [171]. Continuous monitoring also introduces unresolved questions about provider responsibilities for data review, alert response, and timely intervention that current clinical protocols and reimbursement structures do not adequately address, creating liability concerns among healthcare providers. Promising solutions include automated decision support systems that convert complex data into actionable insights; tiered alerting frameworks that filter information by clinical significance; seamless EHR integration standards; targeted training programs for providers; and innovative care models that distribute data management responsibilities across specialized teams rather than overburdening individual clinicians.

### 5.3. Ethical Considerations and Privacy Protection

The deployment of multi-modal biosensing technologies for depression monitoring presents unique ethical challenges that transcend conventional healthcare data concerns. Mental health data represents one of the most sensitive categories of personal information, necessitating specialized frameworks that balance clinical utility with robust privacy protection [172,173].

Contemporary privacy-preserving technologies offer promising solutions to the unique challenges of mental health data protection. Federated learning architectures, exemplified by the FAITH project [174], enable collaborative model development while maintaining data locality, complemented by homomorphic encryption for secure computation and edge computing for local processing [175]. However, these technical approaches must navigate a complex regulatory landscape where HIPAA and GDPR impose stringent requirements that traditional de-identification methods cannot satisfy [176], while emerging standards from IEEE, WHO, and the Digital Therapeutics Alliance emphasize transparency, continuous consent, and recognition that longitudinal depression monitoring generates uniquely sensitive temporal data. Mental health data governance presents distinct challenges: individuals with depression may experience diminished decision-making capacity requiring dynamic consent frameworks, historical data creates vulnerabilities for employment or insurance discrimination [177], and algorithmic complexity raises concerns about interpretability and potential bias [126]. Implementing ethical biosensing systems therefore demands comprehensive strategies integrating privacy-by-design principles (data minimization, purpose limitation), institutional frameworks (specialized review boards, patient advisory committees), and robust technical safeguards (end-to-end encryption, automated retention policies) that balance clinical utility with patient autonomy and dignity [178].

## 6. Conclusions and Future Perspectives

Integration of sensing technologies with therapeutic interventions offers potential for adaptive, personalized treatment delivery based on real-time biosignatures [179]. These emerging closed-loop systems detect physiological and behavioral patterns during vulnerability periods and automatically adjust treatment parameters in response to measured intervention outcomes, thereby supporting more precise management of depression through continuous bidirectional interaction between monitoring and therapeutic components. Early clinical implementations, particularly in deep brain stimulation for treatment-resistant depression, have demonstrated proof-of-concept success where neural biomarkers trigger therapy selectively when symptom severity is elevated, though most applications remain in experimental stages with limited validation across broader populations. The most advanced closed-loop implementations combine electrochemical sensing of neurotransmitters with precision drug delivery or electrical stimulation, enabling personalized intervention optimization through iterative refinement based on individual response patterns.

Intelligent systems incorporating reinforcement learning approaches may eventually enhance treatment efficacy while reducing adverse effects through precision delivery, although significant technical and regulatory challenges remain in translating these approaches from laboratory settings to clinical practice. While current closed-loop systems primarily focus on electrical neuromodulation, next-generation approaches are expanding to include automated pharmacological interventions based on continuous monitoring of depression-relevant neurotransmitters and biomarkers through electrochemical sensing platforms and minimally invasive biosensors. Future developments will likely integrate multiple sensing modalities with various intervention mechanisms to create comprehensive closed-loop systems that can address the biological heterogeneity of depression, potentially transforming management of this complex condition from periodic clinical assessments to continuous, objective, and personalized care.

## Figures and Tables

**Table 1:** Comparison of biosensor technologies for depression monitoring.

Biosensor Type	Target Biomarkers	Strengths	Limitations	Reference
Electrochemical Biosensors	Serotonin; dopamine; norepinephrine; antidepressant	Precise neurotransmitter detection with continuous monitoring capabilities	Complex matrices cause electrode fouling	[21,22]
HRV/ECG Sensors	Heart rate variability; cardiac rhythms; autonomic function	Non-invasive measurement	Multiple confounding factors interference	[45,59]
EEG-based Wearables	Neural oscillations; frontal alpha asymmetry	Non-invasive EEG directly measures	Movement artifacts and spatial resolution limitations	[35,43,60]
Actigraphy/ Movement Sensors	Physical activity; sleep patterns; psychomotor changes	Highly unobtrusive; extended wear time; objective behavioral metrics	Indirect measures; limited specificity for depression; environmental confounds	[50,52,61]
Skin Conductance Sensors	Electrodermal activity; sympathetic nervous system activation	Simple integration into wearables	Environmental influences; low specificity for depression	[58,62]
Multi-modal Integrated Systems	Combined physiological, behavioral, and environmental	Comprehensive assessment	System complexity; increased cost	[17]

**Table 2:** Summary of performance metrics for depression detection systems.

Technology/System	Accuracy	Sensitivity	Specificity	Device Form Factor	Study Setting	Reference
Smartphone + Wearables Integration	89%	87%	93%	Phone + wrist/chest	Real-world	[15]
HRV-based Screening	NR	73.3%	80.6%	Wrist-worn	Clinical	[59]
EEG-based Classification (ML algorithms)	90%	NR	NR	Head-worn (multi-electrode)	Laboratory	[35]

## Data Availability

No new data were created or analyzed in this study. Data sharing is not applicable to this article.
